# Two routes to senescence revealed by real-time analysis of telomerase-negative single lineages

**DOI:** 10.1038/ncomms8680

**Published:** 2015-07-09

**Authors:** Zhou Xu, Emilie Fallet, Camille Paoletti, Steffen Fehrmann, Gilles Charvin, Maria Teresa Teixeira

**Affiliations:** 1 Centre National de la Recherche Scientifique, UMR8226, Laboratoire de Biologie Moléculaire et Cellulaire des Eucaryotes, Institut de Biologie Physico-Chimique, 75005 Paris, France; 2 Sorbonne Universités, UPMC University Paris 06, UMR8226, Laboratoire de Biologie Moléculaire et Cellulaire des Eucaryotes, Institut de Biologie Physico-Chimique, 75005 Paris, France; 3 Institut de Génétique et de Biologie Moléculaire et Cellulaire, 1 rue Laurent Fries, Illkirch, 67400, France

## Abstract

In eukaryotes, telomeres cap chromosome ends to maintain genomic stability. Failure to maintain telomeres leads to their progressive erosion and eventually triggers replicative senescence, a pathway that protects against unrestricted cell proliferation. However, the mechanisms underlying the variability and dynamics of this pathway are still elusive. Here we use a microfluidics-based live-cell imaging assay to investigate replicative senescence in individual *Saccharomyces cerevisiae* cell lineages following telomerase inactivation. We characterize two mechanistically distinct routes to senescence. Most lineages undergo an abrupt and irreversible switch from a replicative to an arrested state, consistent with telomeres reaching a critically short length. In contrast, other lineages experience frequent and stochastic reversible arrests, consistent with the repair of accidental telomere damage by Pol32, a subunit of polymerase δ required for break-induced replication and for post-senescence survival. Thus, at the single-cell level, replicative senescence comprises both deterministic cell fates and chaotic cell division dynamics.

## Abstract (web summary)

Erosion of telomeres eventually causes replicative senescence, but mechanisms underlying the variability and dynamics of the pathway are not known. Here, the authors examine senescence in single yeast cells with inactivated telomerase to reveal two mechanistically distinct routes to senescence.

## Graphical abstract

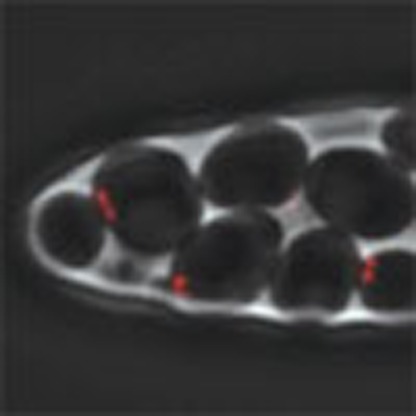

The reverse transcriptase telomerase counteracts the loss of telomere sequences during eukaryotic DNA replication. In human somatic cells, which generally lack telomerase, telomere shortening eventually causes replicative senescence and thus serves as a mechanism to limit cell division and prevent uncontrolled proliferation, as, for example, in cancer^1^,^2^. Current models suggest that when one or several telomeres reach a critical length, they lose the protective cap and expose naked DNA, thereby activating a DNA damage checkpoint pathway that results in cell-cycle arrest^3^,^4^. In mutant *Saccharomyces cerevisiae* lacking telomerase, gradual telomere shortening eventually leads to a similar replicative senescent state^5^,^6^. Some rare cells may overcome senescence by elongating telomeres through either reactivation of telomerase or alternative recombination-based mechanisms^7^,^8^. In mammals, such variants are precursors of cancer cells. Therefore, elucidating the mechanisms underlying the establishment of senescence may shed light on the relationship between telomere dysfunction and carcinogenesis^9^.

Replicative senescence is an intrinsically heterogeneous process. In *S. cerevisiae*, cell-to-cell variations in cell-cycle duration and generation number are influenced by many factors, including the initial telomere length distribution (more precisely, the initial length of the shortest telomere^10^,^11^), the stochasticity of the telomere-shortening mechanism^12^ and other less well-characterized events^13^,^14^. This heterogeneity is supported by analyses of single senescent colonies^5^,^14^,^15^; however, methods that allow precise time-resolved analysis of individual cell lineages over multiple generations are still lacking. Such methods would undoubtedly provide a more accurate understanding of the molecular events controlling senescence.

Here we developed a microfluidics device to analyse consecutive cell divisions from telomerase inactivation to cell death and characterize the transition into replicative senescence. We found that the replicative dynamics of individual lineages contrasts sharply with the classical view of a progressive loss of proliferation potential during senescence, and instead reflects an abrupt lengthening of the cell cycle over the course of one or two cell cycles. Unexpectedly, we also discovered that a substantial proportion of lineages did not behave in a manner consistent with canonical telomere-shortening-driven signalling, but instead exhibited intermittent periods of cell-cycle arrest at DNA damage checkpoints followed by resumption of normal cell cycles before finally entering terminal senescence. This behaviour could not be explained by classical models of gradual telomere attrition. Cells with this phenotype persist only at low frequency in bulk cultures, making them undetectable in conventional population-averaged assays. However, they could provide a basis for further genomic alterations with dramatic consequences, such as cancer emergence.

## Results

### A microfluidics device to track senescent cell lineages

**Figure 1:**
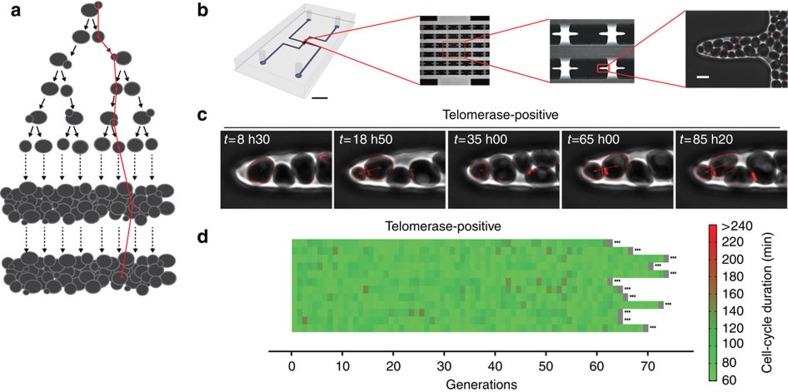
A microfluidics-based approach to the analysis of single lineages. (**a**) Schematic representation of single-lineage tracking (in red). Starting from a single cell, we followed the lineage by tracking one of the two cells after each division, regardless of the daughter/mother cell status. (**b**) Image of the microfluidics chip showing the design of the chambers and microcavities. Scale bars, 5 mm (black) and 5 μm (white). (**c**) Overlays of sequential phase contrast and fluorescence images of a telomerase-positive cell lineage. The Cdc10-mCherry marker at the bud neck (red) allows monitoring of cell-cycle progression and the mother–daughter relationship. (**d**) Display of independent wild-type lineages (yT538, *n*=12). Each horizontal line represents a single lineage, and each segment is a cell cycle. The ellipsis (…) at the end of each lineage indicates that the cell was still alive at the end of the experiment. Cell-cycle duration is indicated by the colour bar.

To characterize and address the cause of cell-to-cell variations in senescence, we have developed a single-cell imaging methodology based on a microfluidic device that allows individual cell lineages to be tracked with high spatial and temporal resolution (Fig. 1a,b)^16^. The microfluidics chip includes two main channels for the flow of medium and an array of central chambers that house the cells of interest (Fig. 1b). Yeast cells are loaded into the small chambers, where they divide and invade microcavities (Fig. 1b,c and Supplementary Movie 1). The cell reaching the tip of the microcavity can then be monitored using phase contrast and fluorescence microscopy. In *S. cerevisiae*, cell division is asymmetric and mother cells can produce only a limited number of daughters. This phenomenon, termed *mother cell ageing*, is observed regardless of telomerase inactivation^16^,^17^. To investigate telomerase-dependent replicative senescence in single dividing cells without interference from mother cell ageing, the tracked cell should have a replicative age low enough to prevent the appearance of ageing phenotypes (typically <5 divisions, Supplementary Fig. 1a,b and Supplementary Movie 1). We therefore used the haploid W303 strain background, which displays an essentially bipolar budding pattern because of a *bud4* mutation^18^. This ensured that the cell at the tip of the microcavity was frequently replaced by its daughter cells. To avoid tracking cells that were ultimately ejected from the microcavity, we selected an individual cell at the point of death (or termination of the experiment) and retrospectively tracked the preceding cell divisions to recreate its entire lineage (see Methods). With this set-up, we were able to monitor single-cell lineages for >70 divisions under physiological conditions (Fig. 1d, Supplementary Fig. 1c and Supplementary Movie 1).

**Figure 2:**
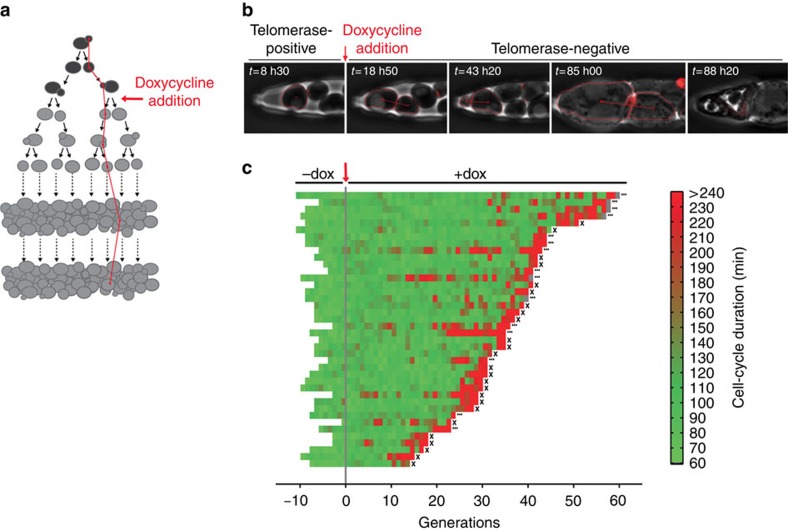
Telomerase inactivation-induced replicative senescence in single lineages. (**a**) Schematic representation of senescence-tracking in the TetO2-*TLC1* strain (as in Fig. 1a). (**b**) Sequential phase contrast and fluorescence images (as in Fig. 1c) of a TetO2-*TLC1* cell lineage. Addition of doxycyline renders the lineage telomerase-negative. (**c**) Display of TetO2-*TLC1* lineages (yT528, *n*=40). Cells were grown in microfluidic chambers for several generations before the addition of doxycycline (30 μg ml^−1^ in the flowing medium) to inactivate telomerase at generation 0. An ellipsis (…) indicates that the cell was alive at the end of the experiment and X indicates cell death. A grey segment indicates that the cell cycle was not complete at the end of the experiment.

To ensure complete temporal control over telomerase inactivation, we used a doxycycline-repressible *TLC1* gene encoding telomerase template RNA (TetO2-*TLC1*). In the absence of doxycycline, lineages with active telomerase showed no sign of mortality (Fig. 1c,d). However, after telomerase repression by the addition of doxycycline, TetO2-*TLC1* cells underwent a limited and highly heterogeneous number of divisions before cell death (37±12 (median±s.d.); coefficient of variation (CV))=0.32; Fig. 2a–c and Supplementary Movie 2 and 3). To determine whether the initial telomere length distribution contributed to this variability, we analysed clonal populations (in which the initial cell starts with a unique telomere length distribution) of a telomerase-inactive strain (described below). This strain displayed significantly smaller variations in division number before lysis (CV=0.11 and 0.15 for two clones; Supplementary Fig. 2a,b), suggesting that the heterogeneous response to telomerase loss observed with TetO2-*TLC1* cells was predominantly because of interclonal variations in the initial telomere length distribution.

The proliferative capacity of individual TetO2-*TLC1* cell lineages observed here is lower than that measured in bulk populations of cells, which undergo 40–80 population doublings depending on the strain background and initial mean telomere length^5^,^12^,^19^. We hypothesized that this apparent discrepancy may be due to competition and selection bias intrinsic to bulk cultures (that is, fitter cells outgrow slow-growing or arrested cells), which is absent in our single-cell analyses. To test this, we performed an *in silico* competitive growth assay on the basis of the probability of death and cell-cycle duration extracted from all single-lineage data (Supplementary Fig. 3a,b). This simulation quantitatively recapitulated the growth curves observed experimentally in batch cultures of the same strain (Supplementary Fig. 3c), confirming that the larger number of doublings before senescence observed in population growth assays was a consequence of the selection of fit cells.

### Two distinct phenotypes upon telomerase removal

**Figure 3:**
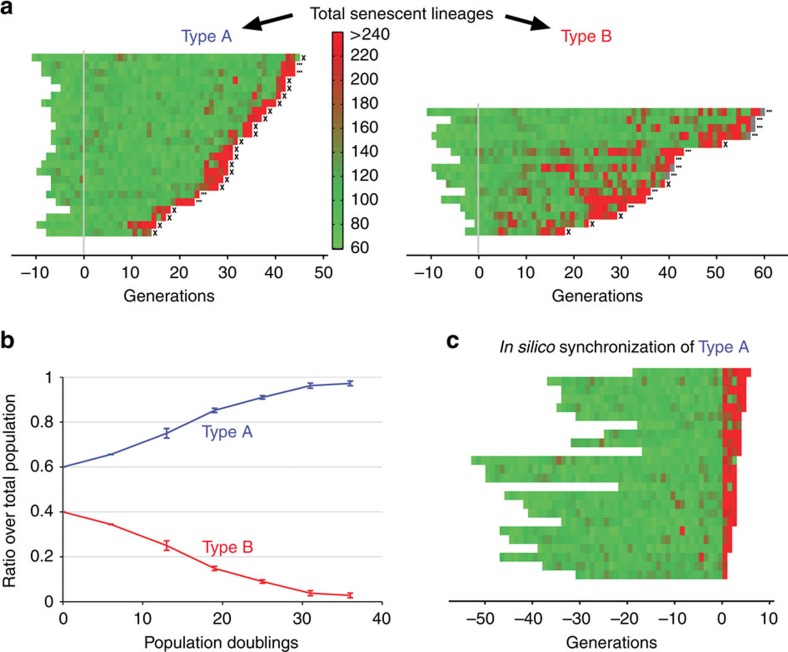
Identification of two routes to senescence. (**a**) The senescent lineages shown in Fig. 2c were clustered according to the absence (type A) or presence (type B) of at least two long cell cycles (exceeding the mean+3 s.d. duration of wild-type cell cycles) before the terminal arrests. (**b**) Simulation of the competition between type A and type B lineages in a virtual bulk culture. The simulation was limited to ∼35 population doublings because of the lack of experimental data in type A lineages beyond this point. (**c**) Type A lineages synchronized *in silico* at the point of transition to replicative senescence, indicated by 0 generation on the *x* axis.

Importantly, our single-cell tracking method allowed us to precisely determine the cell-cycle durations of individual lineages, which cannot be determined from population studies. We found that nearly all TetO2-*TLC1* lineages displayed one or more particularly long cell cycle leading to cell death (indicated in red in Fig. 2c), at which point up to 70% of the cells were arrested in G2/M (Fig. 2b, *t*=85 h), consistent with previous observations of senescence arrest^14^. The longer cell cycles were not associated with the older mother cells, ruling out the contribution of mother cell ageing to replicative senescence in this experimental setting (Supplementary Fig. 1b). Strikingly, a significant fraction of lineages showed a distinct phenotype, with successive long cell cycles occurring well before entry into terminal senescence (Fig. 2c). To quantify this, we defined a significantly long cell cycle as having a duration greater than the mean+3 s.d. of the cycles of telomerase-positive cells. We then clustered the lineages according to the absence (type A, 60% of the lineages) or presence (type B, 40% of the lineages) of at least two long cell cycles before the terminal successive arrests (Fig. 3a). To evaluate the robustness of this threshold-based clustering, we performed two additional independent clustering analyses on the basis of Gaussian distribution mixture models and obtained nearly identical frequencies of type A and type B lineages (Supplementary Fig. 4). To assess the relative competitive potential of type A and B lineages, we simulated their growth in a mixed bulk culture and found that type A lineages rapidly outgrew type B lineages because of their shorter division times (Fig. 3b), even though type B cells had greater median chronological longevity than type A cells (135 (99;+*∞*) and 74 (66;77) h; median (95% confidence interval (CI)), respectively, Supplementary Fig. 5). These data suggest that, whereas type A lineages correspond to the commonly studied ‘senescent cells’, type B lineages represent a ‘cryptic’ phenotype of telomerase inactivation. Despite being present at a significant frequency, the type B lineage is virtually invisible in population studies and can only be revealed in single-cell analyses that eliminate competition bias.

Type A lineages displayed up to 40 cell cycles of duration 1.5±0.3 h (mean±s.d.) before undergoing a sharp transition to one or more long cycles (12±11 h; Fig. 3c and Supplementary Fig. 6). This abrupt change suggests that a single event and/or a threshold effect triggers senescence arrest, which contrasts with the view that senescence involves a gradual decrease in the growth rate, as proposed by studies based on averaging of individual lineages^5^,^14^. Moreover, the transition to slower division and arrest is irreversible in type A lineages, indicating that any attempt at telomere repair fails to reverse the senescence signalling state. Type A lineages would thus be consistent with gradual telomere shortening that goes unnoticed by the cell-cycle checkpoints until one or several telomeres reach a critically short length and the cell cycle is irreversibly arrested.

In contrast, the early cell-cycle delays observed in type B lineages are reversible, as indicated by the resumption of normal cycling following one or more precocious long cycles (Fig. 3a). To check whether type B cells are not simply type A cells that have escaped arrest, we compared the time of onset of the first significantly long cell cycle in both cell types and found a significantly earlier onset for type B cells than for type A cells (17.5±13.4 and 30±9.8 divisions, respectively; median±s.d., *P*=0.03, unpaired *t*-test). Moreover, the first long cell cycle was clearly a harbinger of death for type A cells but not for type B cells, as indicated by the number of divisions between the first and last long cell cycles (3.5±1.4 and 23±7.8 divisions for type A and B cells, respectively). In addition, fewer of the cell-cycle arrests experienced by type B cells occurred in G2/M (48% compared with 70%).

Collectively, these data suggest that the type A and B phenotypes may represent distinct mechanistic responses to telomerase inactivation. Although progressive telomere shortening to a critically short length can account for the behaviour of type A lineages, this model cannot easily account for the precocious cell-cycle delays of type B lineages.

### Stochastic telomere defects cause early cell-cycle delays

To gain further insights into the origin of type B early cell-cycle delays, we next asked whether they were caused by the loss of a non-canonical telomerase function^20^. For this, we analysed the *est2-D670A* mutant, which carries a mutation in the reverse transcriptase motif of Est2, the catalytic subunit of telomerase^21^. In the two *est2-D670A* clones analysed, type A and type B lineages were detected in ratios similar to those of the TetO2-*TLC1* strain (Supplementary Figs 2a,b and 6d). These data confirm that the long cell cycles observed in type B cells are due to a telomerase elongation defect and not to the loss of a non-canonical function.

The *est2-D670A* clones, obtained by the spontaneous loss of a complementing plasmid, were selected as single colonies and grown in liquid medium before injection into the microfluidics device. Thus, in these experiments, each lineage started with a single telomerase-negative cell. We concluded that commitment to the type B pathway is not determined by the initial cell or by the initial telomere set but, rather, occurs stochastically. We therefore predicted that a TetO2-*TLC1* strain with longer initial telomeres in an otherwise isogenic background would produce type B lineages with a higher frequency because the delay in type A lineage senescence would provide an extended window of time for type B long cycles to occur. Indeed, we observed a much higher proportion of type B lineages in the strain with longer initial telomeres than in the strain with normal telomere length (∼82% compared with 40%; Supplementary Figs 7a, 8 and 6d), although the kinetics of telomere shortening in the two strains were similar (Supplementary Fig. 7b). Interestingly, the strain with longer telomeres did not show detectable telomere breaks or display unusual telomere attrition kinetics. This observation suggests that such events either do not correlate with type B lineage arrests or occur only in a subset of lineages and in an asynchronous manner, making them extremely difficult to detect in population-based assays. Overall, the results of these experiments support the notion that type B cells arise stochastically and further confirm that type A and B lineages arise through distinct mechanisms.

### Asymmetry and memory in type B lineages

**Figure 4:**
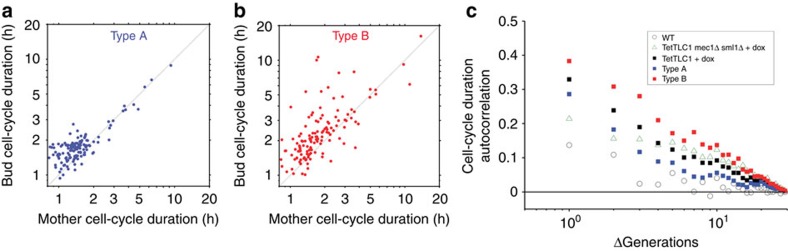
Transmission of the cell-cycle arrest signal at the mitosis and lineage scale. (**a**,**b**) Scatter plots of cell-cycle duration in pairs of sister cells (mother cell and new daughter) in TetO2-*TLC1* (**a**) type A lineages (*n*=141 pairs from three independent lineages) and (**b**) type B lineages (*n*=131 pairs from three independent lineages). Correlation coefficients (95% CI) between the pairs are (**a**) *r*=0.97 (0.96; 0.98) and (**b**) *r*=0.73 (0.64; 0.80). (**c**) Autocorrelation functions of cell-cycle durations in the indicated strains treated with or without doxycycline. Telomerase-negative lineages, particularly type B cells, show stronger autocorrelation than telomerase-positive cells. Mec1 deficiency suppresses memory.

To further investigate the origin of the cell-cycle delays, we compared the cell-cycle durations of pairs of sister cells born from a single mitotic event. The cell-cycle durations in type A pairs showed a strong correlation (Fig. 4a, correlation coefficient (95% CI) *r*=0.97 (0.96; 0.98)), even for the long cycles (*r*=0.98 (0.93; 0.99)). These findings are consistent with senescence being signalled by a single critically short telomere following replication by the semiconservative DNA replication machinery and then being transmitted through mitosis to both cells^10^,^11^,^12^. In contrast, a weaker correlation was observed for cell-cycle durations in type B pairs (Fig. 4b, *r*=0.73 (0.64; 0.80)), particularly for the long cycles (*r*=0.61 (0.40; 0.77)), further arguing that these cycles occur stochastically. We next asked whether the first precocious long cell cycle dictates the duration of subsequent ones by keeping a ‘memory’ of the initial duration. For this, we calculated the autocorrelation function of cell-cycle duration in the lineages^22^. Whereas wild-type cells had an autocorrelation function showing no memory of cell-cycle duration over divisions (timescale *τ*=0.6 (0.5; 0.7) divisions), telomerase-negative lineages had a specific cellular memory (*τ*=1.5 (1.2; 2.0) divisions; Fig. 4c). Furthermore, only type B lineages contributed to this autocorrelation (*τ*=3.2 (2.5; 4.5) divisions), whereas type A lineages were more comparable to wild-type cells and exhibited no significant memory (*τ*=1.0 (0.9; 1.3) division). Thus, type B cells are fundamentally imbued with a long-term memory of previous cell-cycle durations. Taken together, our results suggest the transmission through mitosis of a marker that is generated stochastically in the absence of telomerase, signals cell-cycle delay and increases the probability of additional long cell cycles in type B, but not type A, lineages.

### DNA damage checkpoint activation in senescence

**Figure 5:**
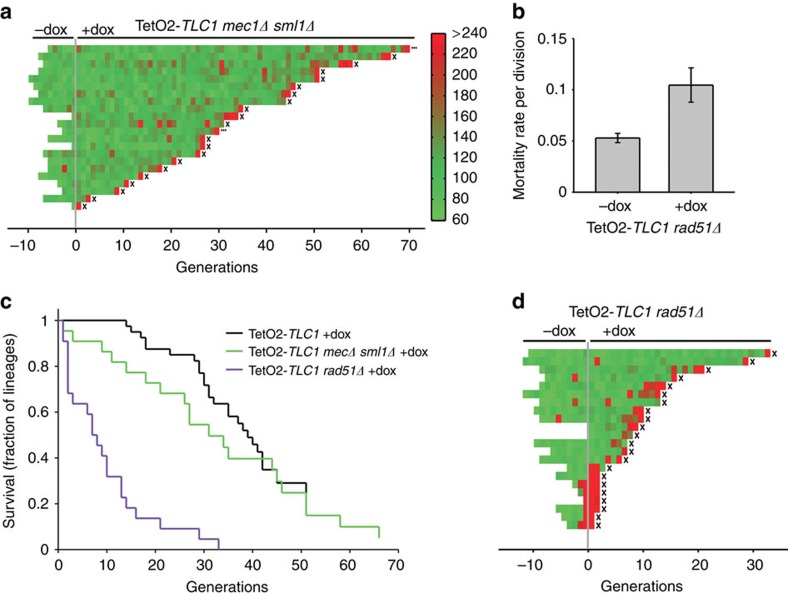
DNA damage checkpoint and homologous recombination shape senescence dynamics. (**a**) Analysis of telomerase-negative *mec1Δ sml1Δ* lineages (yT598, *n*=22). (**b**) Mortality rate per division of *RAD51* mutants with or without telomerase activity (± doxycycline). See Supplementary Fig. 9d for calculation of mortality rates. Error bars represent 95% CI. (**c**) Survival curves of the indicated telomerase-inactive strains. (**d**) Analysis of telomerase-inactivated *rad51Δ* lineages (yT641, *n*=22).

To determine whether this marker is related to DNA damage, we deleted the major DNA damage checkpoint kinase, Mec1/ATR, which is also involved in signalling short telomeres in the absence of telomerase in budding yeast^11^,^14^,^15^. This was performed in a *sml1Δ* background to suppress the lethality of *MEC1* deletion^23^. Indeed, *MEC1 SML1* deletions abolished senescence-specific ‘memory’, as indicated by the autocorrelation function of doxycycline-treated TetO2-*TLC1 mec1Δ sml1Δ* cells (*τ*=0.9 (0.6; 1.4) divisions, Fig. 4c). Moreover, while *SML1* inactivation suppressed many precocious long cell cycles, loss of Mec1 further reduced the frequency of both precocious and terminal *consecutive* long cell cycles, which no longer exhibited extended G2/M phases (Fig. 5a and Extended Data Supplementary Fig. 9a,b), indicating that early cell-cycle delays in type B cells indeed stemed from DNA damage. As early cell-cycle delays were not observed in the presence of telomerase elongating activity, these results indicate that the delays are caused by premature telomeric damages sensed by the DNA damage checkpoint pathway.

### Impact of Rad51 on cell viability and telomere maintenance

Type B cells are able to resume normal cell cycles after slower divisions, suggesting that following damage and checkpoint activation, cells may undergo adaptation^24^ or recover after the damage is repaired. We thus evaluated the contribution of homology-directed repair (HDR), which is known to act at telomeres in the absence of telomerase^6^,^7^,^11^,^25^,^26^,^27^,^28^. Deletion of *RAD52*, a pivotal HDR factor, caused most lineages to die within 10 divisions in our microfluidics assay, regardless of the presence of active telomerase. This precluded a quantitative analysis of the contribution of Rad52 to senescence. Loss of the recombinase Rad51 also dramatically increased the rate of spontaneous cell death (compare Supplementary Fig. 9c with Fig. 1d), albeit to a lesser extent than Rad52 deficiency. The survival curve of active telomerase-expressing *rad51Δ* cells decays exponentially, indicating that they are subject to stochastic death events with a constant mortality rate of ∼5% per division (Fig. 5b and Supplementary Fig. 9d). This is probably due to genomic instability^29^ and, notably, only slightly decreases overall growth in bulk liquid culture (Supplementary Fig. 3c). Although Rad51 associates with the shortest telomeres^25^,^30^ and has genome-wide effects in the presence of telomerase, its contribution to cell viability early after telomerase inactivation remains elusive. Here our single-lineage analyses show that upon telomerase inactivation, most *rad51Δ* lineages died within 10 divisions, at which time telomeres had shortened by only ∼30 bp (Fig. 5c,d, Supplementary Fig. 7c and Supplementary Movie 4). Inactivation of telomerase in the *rad51Δ* mutant increased mortality from ∼5 to ∼11% per division (Fig. 5b,d and Supplementary Fig. 9d), whereas telomerase inactivation in Rad51-positive cells had no effect on mortality during the first 10 divisions (Fig. 5c). Thus, the increase in mortality reveals a synergy between Rad51 and telomerase in the protection of telomeres that is distinct from the genome-wide role of Rad51. Furthermore, the exponential decay in the survival curve indicates that, in cells lacking both Rad51 and telomerase, death is a stochastic and age-independent process. Overall, the analysis of single-cell lineages has allowed us to detect and quantify the constitutive contribution of homologous recombination to telomere protection, which presumably occurs by preventing or rescuing telomere break events, as previously proposed^31^.

### Type B lineages are suppressed in the absence of Pol32

**Figure 6:**
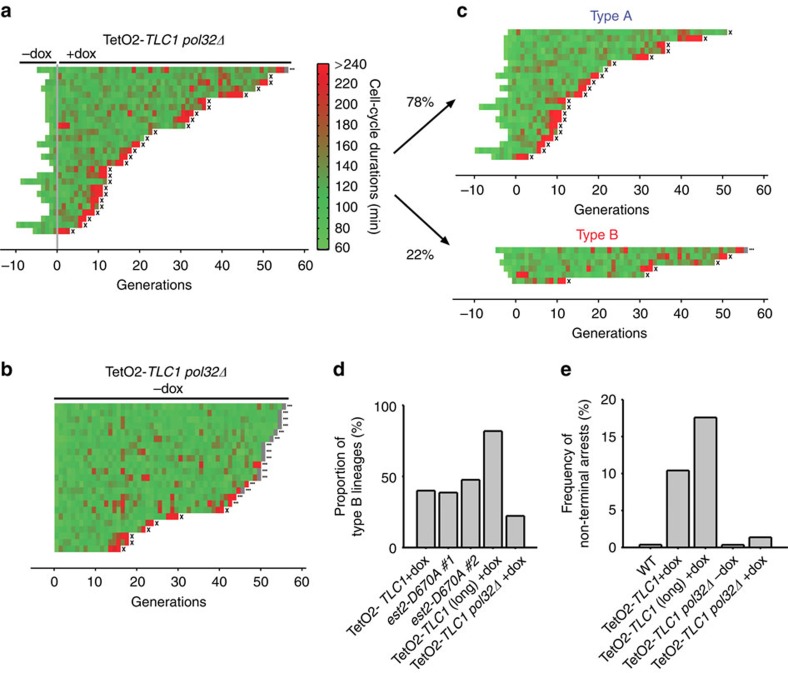
Pol32 is required for type B lineages. (**a**) Analysis of telomerase-negative *pol32Δ* lineages (yT662, *n*=27). (**b**) Analysis of telomerase-positive *pol32Δ* lineages (yT662, *n*=23). (**c**) Telomerase-negative *pol32Δ* lineages were clustered into type A and type B lineages. (**d**) Quantification of the proportion of type B lineages in the indicated strains. The ‘TetO2-*TLC1* (long) +dox’ strain corresponds to the strain with longer initial telomeres shown in Supplementary Fig. 8 and Supplementary Fig. 7a, lanes 3 and 4, which displayed a higher frequency of type B lineages. (**e**) Quantification of the frequency of non-terminal arrests in the indicated strains, used as an alternative assessment of the proportion of type B lineages.

Break-induced replication (BIR) is a HDR pathway in which one end of a broken chromosome can invade a homologous region and prime DNA replication up to the end of the template chromosome. This pathway prevails at telomeres in the absence of telomerase and relies on Pol32, a nonessential subunit of polymerase δ (refs 32, 33, 34, 35; Fig. 6a,b). In the presence of telomerase, *POL32* deletion increased the mortality rate to ∼0.7% per division and increased the average cell-cycle duration and variability (108±38 min, Fig. 6b and Supplementary Fig. 1c). This result suggests that Pol32 promotes efficient cell-cycle progression, consistent with its role in BIR. Cells in which both telomerase and Pol32 were inactivated underwent 17.5±16 divisions (median±s.d.), indicating an acceleration of replicative senescence, as reported previously^19^,^25^ (Fig. 6a). This acceleration was not due to differences in telomere length or in telomere-shortening kinetics (Supplementary Fig. 7d). We used cluster analysis to separate *pol32Δ* senescent lineages into types A and B, adapting the threshold for significantly long cell cycles (mean+3 s.d.≈220 min) to account for the longer average cell-cycle duration observed in the telomerase-positive *pol32Δ* cells (Fig. 6c and Supplementary Fig. 1c). Strikingly, the absence of Pol32 reduced the proportion of type B lineages from 40 to ∼22% (Fig. 6c,d). This ratio was probably an overestimation because some of the observed arrests may have occurred regardless of the lack of telomerase (compare Fig. 6b,c). Indeed, the overall frequency of non-terminal arrests in *pol32Δ* telomerase-negative and -positive lineages were ∼1.4% and ∼0.4%, respectively (Fig. 6e), indicating that ∼26% (0.4/1.4) of the non-terminal arrests in the telomerase-negative lineages were due to the deletion of *POL32*, regardless of the absence of telomerase activity. However, because replicative senescence was accelerated in the *pol32Δ* strain, we reasoned that the reduced proportion of type B lineages might reflect the lack of opportunity for precocious arrests rather than a mechanistic suppression of type B lineages. We thus calculated the frequency of non-terminal arrests in telomerase-negative *POL32* and *pol32Δ* strains as an alternative method of assessing type B lineage frequency independently of the length of the lineages. We found that this frequency was ∼10% in telomerase-negative *POL32* and ∼1.4% in telomerase-negative *pol32Δ*, arguing in favour of a genuine suppression of the early arrests occurring in type B lineages (Fig. 6e). Therefore, our data strongly suggest that Pol32 contributes to the formation of type B lineages and that BIR may be involved in the repair and recovery of the precocious damage. In the absence of Pol32-dependent repair, type B-specific damage would lead to cell death, making the type A and B lineages indistinguishable and thus accounting for the observed acceleration of replicative senescence. Nonetheless, we cannot exclude the possibility that Pol32 may also play a minor role in type A lineages; for example, in the few terminal arrests, but the impact of BIR-based repair seems far more important in the type B lineages.

## Discussion

Our single-lineage and time-resolved analysis of senescence has uncovered two distinct phenotypes on telomerase removal. The phenotypes could not have been characterized using conventional population studies, which are based on averages of individual events and favour the selection of the most proliferative lineages. Several lines of evidence demonstrate that the type A and B telomerase-negative lineages occur via biologically distinct routes. First, they follow distinct kinetics to the first cell-cycle delay and to cell death and are separable by modulating the initial telomere length. Second, the early long cell cycles are reversible in type B lineages but not in type A lineages. Third, the DNA damages causing the cell-cycle delays in the two lineages are different both in terms of transmission through mitosis and requirements for Pol32 for repair. Importantly, the behaviour of both type A and type B lineages is inconsistent with a progressive increase in cell-cycle duration as the telomeres shorten.

In type A lineages, the kinetics of the switch into senescence correlates with the first telomere(s) reaching a critically short length(s), suggesting that this may be the senescence trigger. Likewise, the symmetric transmission of the cell-cycle arrest phenotype in mitosis is reminiscent of segregation of a genetic marker such as telomere length. To verify this, we developed a mathematical model of senescence taking into account the molecular mechanism of telomere shortening^12^ and different hypotheses for the senescence onset signal. We found a perfect match between type A experimental data and simulations based on the hypothesis that a single telomere reaching a critically short length triggers an irreversible cell-cycle arrest (Bourgeron *et al.*, unpublished). Furthermore, type A cells undergo a single transition into very long and abnormal cell cycles followed by cell death. Therefore, eroded telomeres seem strongly resistant to repair.

In contrast, type B lineages experience early spontaneous and reversible cell-cycle delays, that often persist in cell lineages, and are inherited in an asymmetrical manner, and are thus inconsistent with gradual telomere erosion. While the molecular mechanisms causing the first arrest in type B lineages remain unclear, the cells seem to keep a memory of this event, which is suppressed by the absence of Mec1 and Sml1. Several non-mutually exclusive hypotheses are consistent with this phenomenon: (i) the telomeres may be in a partially unprotected state that stochastically signals an arrest in subsequent cell cycles, (ii) the cells may undergo adaptation^24^,^36^, thus proceeding through a few divisions before arresting again because the initial damage has not been repaired, (iii) extrachromosomal markers, such as telomeric ssDNA circles found in post-senescence survivors^37^, may be generated and poorly segregated or (iv) the initial telomere damage may be repaired by a BIR or another recombination mechanism but generates genetic or telomeric instabilities that can promote more telomeric or genome-wide damage. In accordance with this latter scenario, the early delays observed in type B lineages are suppressed by telomerase elongation activity itself and by the absence of Mec1, Sml1 or Pol32, suggesting that they correspond to telomeric DNA damages, repaired by BIR. BIR could subsequently lead to the elongation of the damaged telomeres by Y′ subtelomeric elements^35^, but also, in combination with a checkpoint deregulation or adaptation, can initiate a cascade of genetic instabilities, shown to occur both in yeast and human cells in some circumstances^38^,^39^,^40^.

The finding that the absence of Rad51 and Rad52 has a strong effect on the viability of all telomerase-negative lineages suggests that these factors may act constitutively to protect telomere replication under conditions of replication stress^41^,^42^. An alternative, but not mutually exclusive, possibility is that the recombination factors could act by preventing the accumulation of ssDNA and signalling by the shortest telomere(s)^25^. In addition, Rad51 and Rad52 could contribute in a Pol32-independent manner^19^ to repair the stochastic telomeric damage in type B lineages in combination with BIR mechanisms. Such damage could be a consequence of the observed replication stress at telomeric repeats, for example^25^,^31^,^43^,^44^,^45^,^46^.

Taken together, our results are consistent with a model in which telomere replication is continuously protected by HDR pathways and by BIR when rare accidental breaks occur. Type A lineages would avoid such breaks, whereas type B lineages would experience an erratic pattern of cell-cycle arrests that contribute to the heterogeneity of senescence. Because of their altered checkpoint state and their dependence on Pol32, type B lineages may accumulate mutations and experience cascades of genome rearrangements, eventually leading to the emergence of genomic variants^33^,^38^,^39^,^40^,^47^,^48^,^49^. Finally, the cryptic status and extended longevity of type B lineages may allow them to re-emerge in long-term cultures and give rise to post-senescent survivors. We speculate that in humans similar type B lineages could well be the precursors of cancer cells in aged tissues.

## Methods

### List of strains

All of the strains used in this study had a W303 background and were corrected for the *rad5–535* mutation, which is naturally present in the W303 strain. See Supplementary Table 1.

### Growth assay

To measure the proliferation potential of telomere-positive and telomere-negative cells, we diluted a log-phase culture of TetO2-*TLC1 RAD51* (yT528) or TetO2-*TLC1 rad51Δ* (yT641) strains to OD_600 nm_=2.3 × 10^−5^ in YPD medium and grew them at 30 °C for 24 h until OD_600 nm_ reached 1.5. The cultures were then divided and grown with or without 30 μg ml^−1^ doxycycline to repress *TLC1*. Each day, the same procedure was applied with an adjustment of the dilution ratio in the telomerase-inactivated cultures in order to reach 0.6<OD_600 nm_<2, after 24 h, to account for the loss of proliferation potential. For monitoring the kinetics of telomere shortening, a similar growth assay was performed except that the cultures were diluted daily to OD_600 nm_=0.01 in YPD+doxycycline (30 μg ml^−1^) and the cells were collected after 24 h of growth.

### Terminal restriction fragment Southern blot

Genomic DNA was extracted from exponential growth cultures using a standard phenol:chloroform:isoamyl (25:24:1) purification procedure and ethanol precipitation. Then, 1 μg of genomic DNA was digested with XhoI and the products were ethanol-precipitated, resuspended in loading buffer (10 mM Tris pH 8.0, 1 mM EDTA, 5% glycerol, 0.04% xylene cyanol FF) and resolved on a 1.2% agarose gel for 14 h at 60 V. The gel was then soaked in a denaturation bath (0.4 M NaOH and 1 M NaCl) for 30 min and transferred by capillarity action to a charged nylon membrane (Hybond XL, GE Healthcare). The telomere-specific oligonucleotide probe (5′-GGGTGTGGGTGTGTGTGGTGGG-3′) was ^32^P-labelled at the 5′ terminus with ATP (γ-^32^P) and T4 polynucleotide kinase (New England Biolabs). The membrane was hybridized using the Rapid-hyb buffer protocol (GE Healthcare). In brief, the membrane was prehybridized at 42 °C in Rapid-hyb buffer for 1 h, then the radioactive probe (20 pmol) was added and the incubation was continued for 1 h. The membrane was washed consecutively with 2 × SSC, 0.5% SDS (42 °C for 10 min); 2 × SSC, 0.1% SDS (42 °C for 20 min); and 0.1 × SSC, 0.1% SDS (20 °C for 30 min). The membrane was then imaged with a Typhoon FLA 9500 scanner (GE Healthcare).

### Telomere elongation

In some experiments (Supplementary Figs 2 and 8), the telomeres were elongated before cells were placed in the device, either to replenish telomere repeats subsequently lost during preculture and sample preparation (Supplementary Fig. 2) or to study the effect of longer telomeres (Supplementary Fig. 8). To reversibly elongate telomeres, we grew cells in rich medium (YPD) containing 5% ethanol for ∼80 or ∼160 generations, serially diluting every 3 days^50^. The cells were then transferred to YPD agar plates without ethanol, and at this point the mean telomere length was measured with Southern blot analysis, as described above and shown in Supplementary Fig. 7a. To ensure the recovery of cells from ethanol treatment, we grew cells for at least 40 divisions in medium without ethanol before placing them in the microfluidic device.

### Microfluidic device

The microfluidic mold was fabricated using standard soft lithography techniques as described^16^. To make the chip, we mixed polydimethylsiloxane (PDMS; Sylgard 184) and curing agent in a 10:1 ratio, degassed it with a vacuum pump for 30 min and poured it into the mold. The PDMS was cured by baking at 70 °C for 5 h and then was carefully removed from the mold. A biopsy puncher (1.5 mm, Harris Unicore) was used to create holes for medium flow. The surfaces of PDMS and a glass coverslip (24 × 50 mm) were surface-activated using a plasma cleaner (Diener Electronic, Germany) to covalently bond the two elements. For injection of cells into the device, synthetic complete medium containing 2% glucose (SD) was filtered using a 0.22-μm polyethersulfone filter (Corning) and loaded into the device using a peristaltic pump (IPCN, Ismatec). Cells from a log-phase culture (0.5 OD_600_) were gently injected into the device using a 1-ml syringe. A constant medium flow (28 μl min^−1^) was maintained throughout the experiment. Control experiments validating the flow rate, medium diffusion into the cavities, nutrient uptake and physiological cell growth were performed as previously described^16^. For experiments with strains expressing the TetO2-*TLC1* construct, cells were allowed to divide and invade the cavities for 12–24 h before the medium was switched to SD containing 30 μg ml^−1^ doxycycline. For experiments with the *est2-D670A* mutant strain (yT639), a clone without the plasmid complementing *EST2* deficiency (pVL291 encoding *EST2* with a *URA3* marker) was selected. Because selection required 20–30 cell divisions, the telomeres were elongated by ethanol treatment before the experiments (see above, Supplementary Figs 2 and 7a). To select a single clone, cells were plated on YPD for 24 h and individual colonies were picked and grown on selecting medium (SD-uracil) to verify plasmid loss (the frequency of spontaneous loss was 5–8%). After an additional 24 h, a colony without plasmid was precultured overnight in SD medium and injected into the microfluidic device as described above. An aliquot of the same culture was analysed using Southern blot analysis to measure telomere length (Supplementary Fig. 7a).

### Time-lapse microscopy

Cells in the microfluidic device were imaged using a fully motorized Axio Observer Z1 inverted microscope (Zeiss), with constant focus maintained with focus stabilization hardware (Definite focus, Zeiss). To minimize phototoxicity, we used light-emitting diode (LED) light sources for both phase contrast and fluorescence images (Colibri 2, Zeiss) with the following parameters: 4.0 V–70 ms for phase contrast and 15% of maximum intensity −400 ms with 2 × 2 binning for the 560 nm LED. The temperature was maintained at 30 °C with a controlled heating unit and an incubation chamber that held the entire microscope base, including the stage and the objectives. Images were acquired every 10 min using AxioVision 4 (Zeiss). All aspects of image acquisition were fully automated and controlled, including temperature, focus, stage position and time-lapse imaging. Images were acquired for >120 h in standard experiments and up to 240 h in the experiment using cells with elongated telomeres.

### Image analysis and single-cell lineage tracking

A custom software written in Matlab, phyloCell 2.1, was used to segment and track cells and to assign mother–daughter links^16^. Time-lapse images were exported as high-resolution TIF files and analysed directly with the graphical user interface of phyloCell. To follow cell-cycle progression and determine the mother–daughter relationship, cells expressed a fluorescent Cdc10-mCherry fusion protein that localized to the septin ring from bud formation to cytokinesis. This fluorescent marker was also used to determine the cell-cycle stage based on the morphology and size of the bud. For details about the routines and algorithms implemented in phyloCell, see ref. 16. In contrast to ref. 16, the cell at the tip of the cavity would frequently be replaced by its daughter cell, which was intended in our approach. To efficiently track such lineages, in which we frequently switched focus from a given cell to its daughter cell, the time-lapse images were retrospectively analysed starting from the last image. This avoided tracking of lineages in which the cells were ejected from the microcavity.

### Computational and statistical analyses

All computational and statistical analyses were performed in Matlab. The functions developed in this study are available on request. For cluster analysis of telomerase-negative lineages, two independent criteria were used: the number of long cell cycles (greater than the mean+3 s.d. of wild-type cell cycle, which may be different from strain to strain) per lineage and the number of transitions per lineage. A transition was defined as two consecutive cycles with a difference in their duration greater than a threshold *S*. The number of transitions per lineage was calculated for *S*=60, 70, 80, 90 and 100 min and averaged for each lineage. For each of these two criteria, we created a mixture model with univariate Gaussian distributions and performed a cluster analysis of the mixture, which defined clusters as data points belonging most probably to the same distribution. The *gmdistribution* class and related methods of the statistics toolbox of Matlab were used for this analysis.

### Autocorrelation function

The normalized autocorrelation function *A*(*k*) for lag *k* was calculated according to^22^:




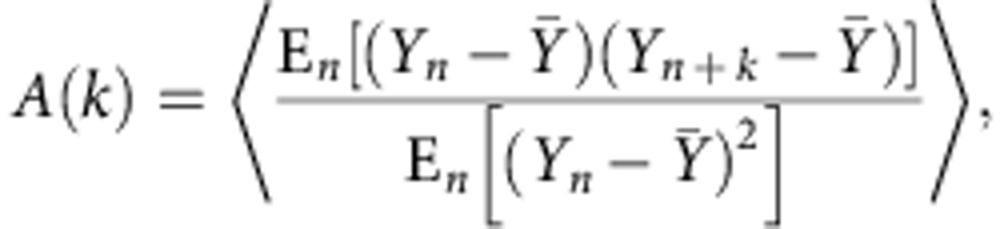




where *Y*_*n*_ denotes the cell-cycle duration at division *n* normalized over the wild-type mean for mother or newborn daughter cell accordingly; 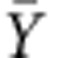 denotes the mean of *Y* over all time points and cells; E_*n*_[] denotes the expected value operator over divisions; and 〈〉 denotes the average over all cells. The autocorrelation function *A*(*k*) measures the similarity between two cell-cycle durations as a function of the time lag *k* between them. For a given strain or condition, for any *k*, *A*(*k*) was averaged over all cell cycles of all lineages. The function was fitted with an exponential function 
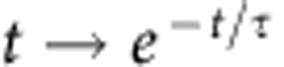
 to obtain an estimate of the autocorrelation timescale *τ* of the process. Typically, *A*(*k*) rapidly drops to 0 after several cell cycles, and the timescale *τ* of this decrease indicates how fast the information of a cell-cycle duration is lost.

### Simulation of competitive culture

To compare cell growth in the microfluidic chip and conventional bulk culture, we numerically simulated a virtual culture in which the analysed lineages would be in competition. From the single-lineage data (Fig. 2c), we extracted the probability of death and the cell-cycle duration landscape (Supplementary Fig. 3b) and used them as inputs to simulate competitive growth (that is, the total number of cells in the virtual culture as a function of time). The simulation of a telomerase-negative culture was performed with a custom automaton developed in Matlab. All parameters were directly derived from the independent lineage data (Supplementary Fig. 3b), and no arbitrary external parameters were used. First, for each generation *n* after telomerase loss, a probability of death 
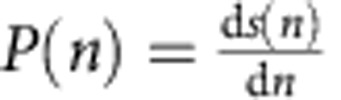
, where *S* is the survival curve, was derived from the fraction of total lineages that ended at this generation. Given the discrete nature of the data, *P* was fitted with a Gaussian function. Second, we calculated a matrix *M*(*n, p*) containing the probability that a cell at generation *n* will divide in *p* units of time (that is, 10 min, which is the temporal resolution of the time-lapse experiments). The number of rows in *M* is restricted to the maximum number of generations undergone by a lineage. *M* is calculated by normalizing the experimental frequency two-dimensional histogram of cells dividing at generation *n* in *p* units of time, shown in Supplementary Fig. 3b. The simulation included dilution steps to mimic those required in experimental senescing cultures. The dilution ratio and the number of cells picked to continue the virtual culture had no detectable effect on the result (data not shown). The automaton starts with 1,000 cells, each of which is randomly assigned a cell-cycle duration chosen from *M*(1, [1, *max*]), that is, the first row of M. For each time point, we determine whether a cell has completed its cycle and then test whether this cell, which is at generation *n*, will die, given *P*(*n*). If it dies, that cell is removed from the array. If not, it divides into two cells (a new cell is created in the automaton), each of which is assigned a new cell-cycle duration corresponding to the generation *n*+1, chosen from *M*(*n*+1, [1, *max*]). To smooth the discrete data, we pick *p* from *M* and account for the variability of biological processes by adding noise according to the CV experimentally observed. This simulation is performed over 60 time points, corresponding to 10 h. The total number of cells is recorded. Then, 1,000 cells are randomly picked from this virtual culture and are run in a second round of simulation (dilution). After 13 rounds, all data are collected and converted into a plot, as shown in Supplementary Fig. 3a. For estimation of the fraction of type A and type B cells in a bulk culture (Fig. 3b), two cell-cycle duration matrices *M*_A_ and *M*_B_ were derived from the two sets of lineages. The automaton was slightly modified to accommodate the simultaneous use of both matrices. The automaton starts with 1,000 cells, as for the standard simulation, but a tag is added to each cell to indicate its type. The initial ratio was 60:40% of type A:type B cells (Fig. 3b). When a cell divides, its type is inherited and the next cell-cycle duration is extracted from either *M*_A_ or *M*_B_. The number of type A or type B cells is recorded at each round of simulation. The simulation is not performed over more than ∼35 population doublings because of the lack of experimental data in type A matrix *M*_A_ beyond this point.

## Additional information

**How to cite this article:** Xu, Z. *et al.* Two routes to senescence revealed by real-time analysis of telomerase-negative single lineages. *Nat. Commun.* 6:7680 doi: 10.1038/ncomms8680 (2015).

## Supplementary Material

Supplementary Information Supplementary Figures 1-9 and Supplementary Table 1.

Supplementary Movie 1 Time-lapse phase contrast images of the telomerase-positive strain (yT538). The cells, loaded in the chamber, divide and invade the cavity. The cell at the tip of the cavity is then monitored over successive divisions.

Supplementary Movie 2 Time-lapse overlay of phase contrast and fluorescence (Cdc10-mCherry) images of a representative type A lineage. The TetO2-TLC1 strain (yT528) was grown in the microfluidic device for ~19 h before doxycycline addition and monitored for >87 h.

Supplementary Movie 3 Time-lapse overlay of phase contrast and fluorescence (Cdc10-mCherry) images of a representative type B lineage. The TetO2-TLC1 strain (yT528) was grown in the microfluidic device for ~19 h before doxycycline addition and monitored for >150 h

Supplementary Movie 4 Time-lapse overlay of phase contrast and fluorescence (Cdc10-mCherry) images of a representative TetO2-TLC1 rad51? lineage (yT641). The cells were grown in the microfluidic device for ~23 h before doxycycline addition and monitored for >64 h.
