# Supplementary material for: Two routes to senescence revealed by real-time analysis of telomerase-negative single lineages
Source: Nat Commun. 2015 Jul 9;6:7680. doi: 10.1038/ncomms8680 (PMC4503340; doi:10.1038/ncomms8680)
Supplement: Supplementary Information — Supplementary Figures 1-9 and Supplementary Table 1. [file ncomms8680-s1.pdf]

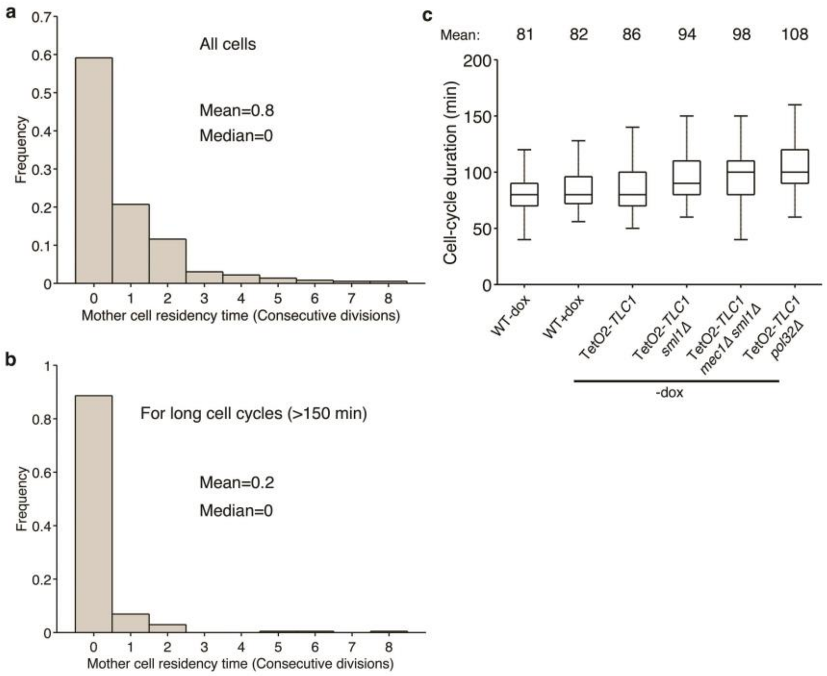

Supplementary Figure 1. Physiological cell division in the microcavities, with no interference from the mother cell ageing phenomenon. a, Frequency of the residency time of a mother cell in a microcavity, showing that the analysed cells have a very low replicative age (essentially <5) compared to the median value for mother cell ageing (≈32[16]). The exponential distribution indicates that the budding pattern in the haploid W303 background is random. **b,** Same as **a,** except the analysis is restricted to cells with long cell cycles (>150 min), showing that nearly all of the analysed cells are new-born daughter cells or young mother cells. **c,** Boxplot of cell-cycle durations of the non-senescent strains used in this study. The central mark in each box is the median, the edges are the 25th and 75th percentiles, and the whiskers are the 95th percentiles. WT-dox, wild-type cells (yT538, *n* = 792); WT+dox, wild-type cells with added doxycycline (30 µg/ml; n = 174); TetO2-*TLC1*, cells expressing the TetO2-*TLC1* construct (yT528, *n* = 239); TetO2-*TLC1 sml1*Δ, TetO2-*TLC1* cells with a deletion of *SML1* (yT599, *n* = 208); TetO2-*TLC1 mec1*Δ *sml1*Δ, TetO2-*TLC1* cells with deletions of both *mec1*Δ *and sml1*Δ (yT598, *n* = 107); TetO2-*TLC1 pol32*Δ, TetO2-*TLC1* cells with a deletion of *POL32* (yT662, *n* = 934). The mean cell-cycle durations are indicated at the top in minutes.

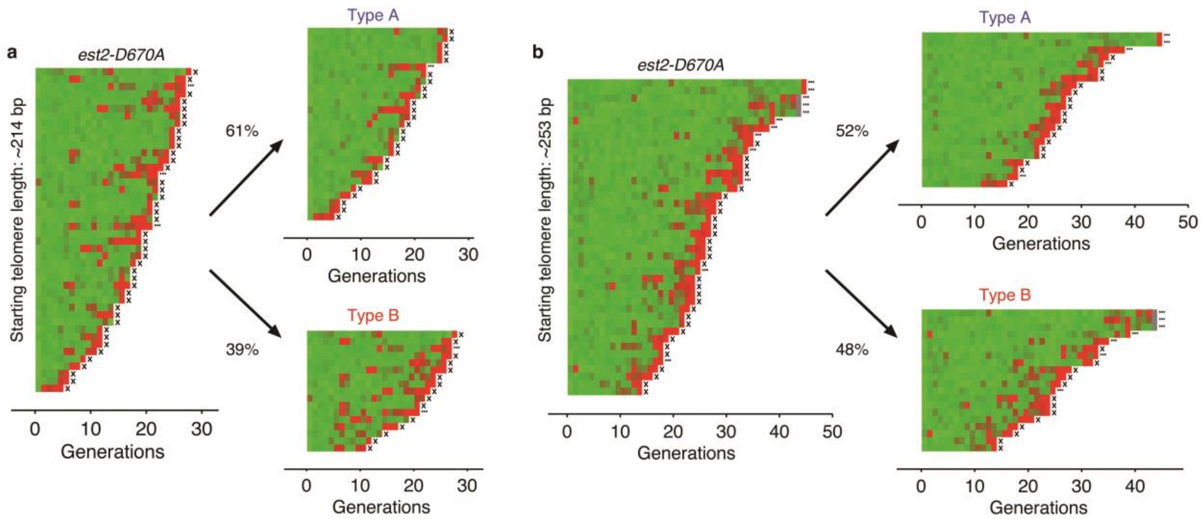

Supplementary Figure 2. Single-lineage analysis of the telomerase mutant *est2-D670A* strain (yT639). a–b, The *est2-D670A* strain, complemented with a plasmid expressing a functional *EST2* gene (pVL291), was treated with ethanol to elongate the telomeres (Supplementary Fig. 7a, lane 6). Two clones were then selected in YPD medium for spontaneous loss of the complementing plasmid. After clonal expansion (~40 and ~30 population doublings for **a** and **b**, respectively), the cells were injected into the microfluidic device, and single lineages were tracked (n = 44 and n = 42 for **a** and **b**, respectively). The proportion of type A and B cells in both clones is indicated.

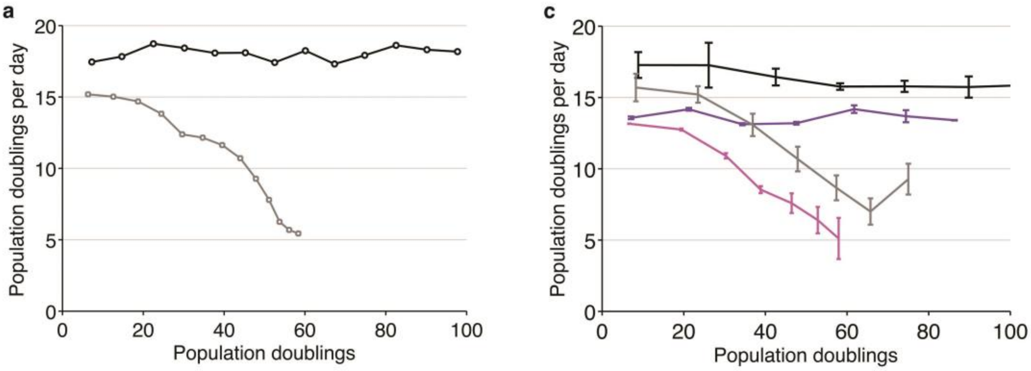

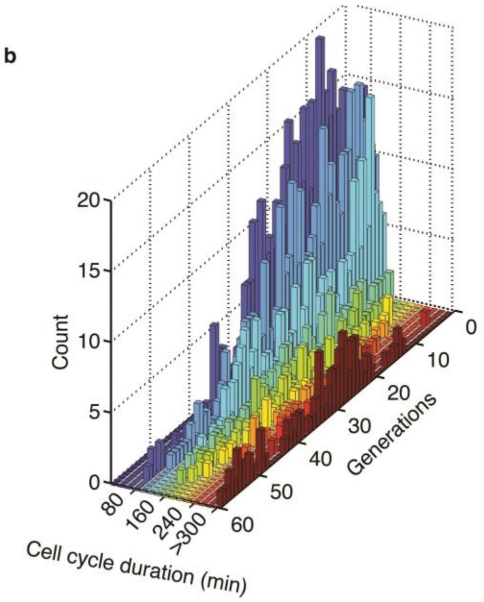

Supplementary Figure 3. Simulation of competitive growth using competition-free lineage data. a, Simulation of the competitive growth of all lineages shown in Fig. 2c. The black and grey lines indicate telomerase-positive and telomerase-negative virtual cultures, respectively. **b,** 2D histogram representing the distribution of different cell-cycle durations over generations. Most cell cycles are distributed around ~90 min in the early divisions. Over generations, the fraction of rapidly dividing cells (blue) decreases as that of slowly dividing cells (red) increases. **c,** Growth of experimental liquid cultures of the TetO2-*TLC1* strain (yT528) with (grey line) or without (black line) doxycycline (30 µg/ml) and of the TetO2-*TLC1 rad51*Δ strain (yT641) with (pink line) or without (purple line) doxycycline (30 µg/ml).

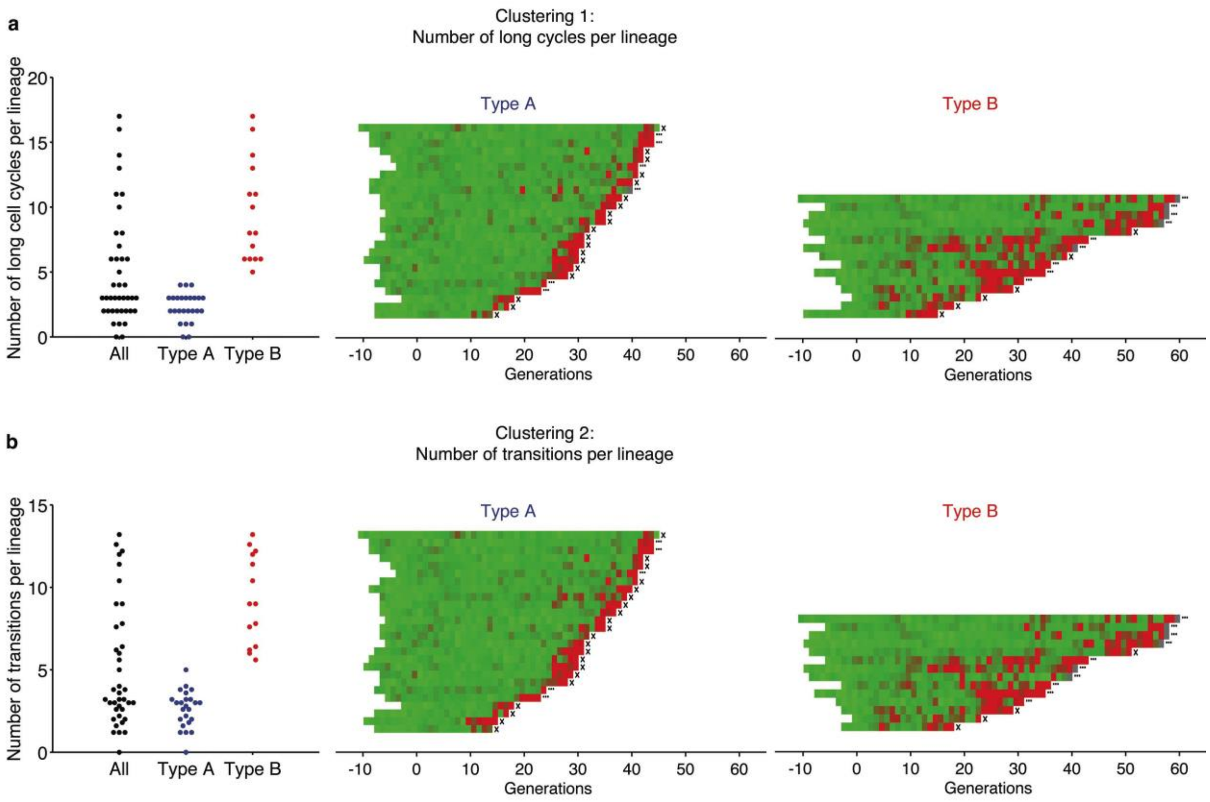

Supplementary Figure 4. Two independent cluster analyses of the telomerase-negative lineages shown in Fig. 2c. a, Clustering using the number of long cell cycles per lineage as a criterion. Left plot: Clustering into type A and B based on a Gaussian mixture distribution model. Right plot: Display of the corresponding type A and B lineages. **b,** Same as **a,** except the number of transitions per lineage was used as a criterion. Note that these two cluster analyses result in type A and B groups that are nearly identical to that shown in Fig. 3a.

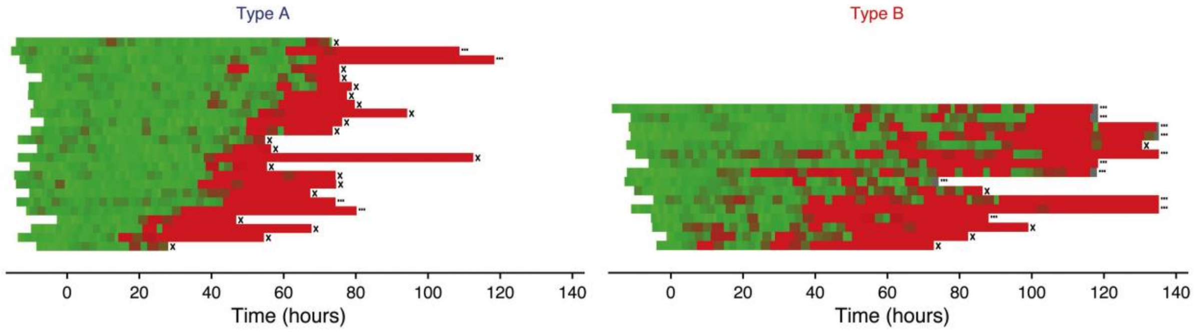

Supplementary Figure 5. Chronological representation of type A and type B lineages, using time as the x-axis. The duration of each cell cycle (each segment) is indicated by both the colour and the length of the segment. The median chronological longevity with 95% confidence interval of type A and B lineages can be calculated using the Kaplan-Meier estimator with censoring: 74 [66;77] and 135 [99;+∞] h, respectively.

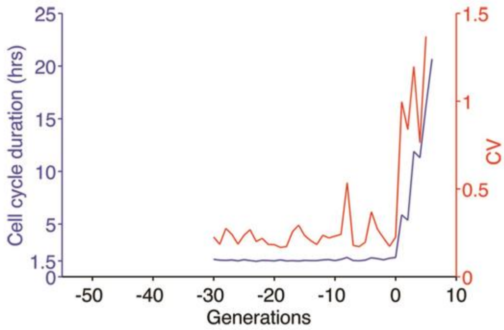

Supplementary Figure 6. Quantification of cell-cycle duration of *in silico* synchronised type A lineages. The mean (blue) and CV (red) of cell-cycle durations for all *in silico* synchronised lineages are shown. The CV increases in parallel with the cell-cycle duration at the transition to senescence (0 on the x-axis), indicating the loss of cell-cycle regulation.

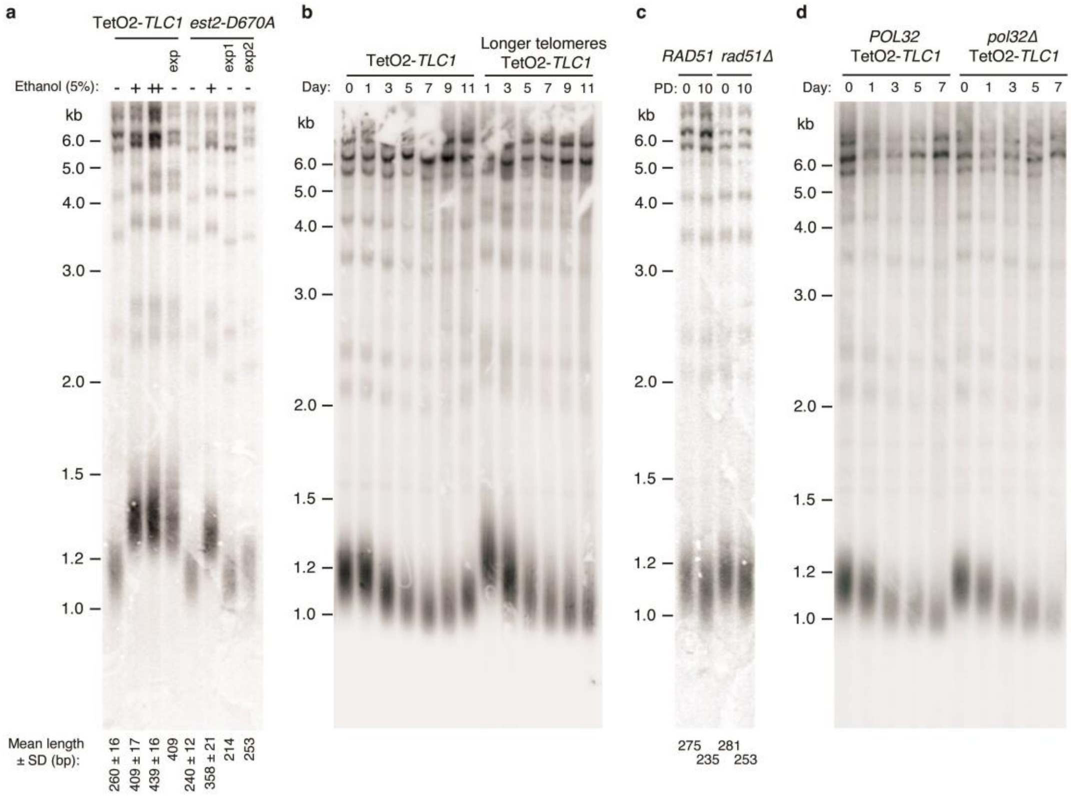

Supplementary Figure 7. *Xho* I terminal restriction fragment Southern blot analyses of Y′ telomeres. a, Telomere lengths of TetO2-*TLC1* (yT528) and *est2-D670A* (yT639) strains. Lanes 1 and 5: untreated strains. Lanes 2, 3, and 6: strains treated with ethanol for ~80, 160, and 80 population doublings, respectively. Lane 4: sample from the experiment shown in Supplementary Fig. 8. Lanes 7 and 8: samples from experiments shown in Supplementary Fig. 2a, b. The mean lengths of the telomeric repeats are indicated below the lanes. Results for multiple ($n \geq 3$) independent experiments are shown as the mean ± SD. **b,** Telomere lengths of telomerase-negative (doxycycline-treated) TetO2-*TLC1* with normal or longer initial telomeres on different days of culture. **c,** Telomere lengths of telomerase-negative (doxycycline-treated) TetO2-*TLC1 RAD51* and TetO2-*TLC1 rad51*Δ cells (yT528 and yT641, respectively) at 0 and 10 population doublings (PD). **d,** Telomere lengths of telomerase-negative (doxycycline-treated) TetO2-*TLC1 POL32* and TetO2-*TLC1 pol32*Δ cells (yT528 and yT662, respectively) on different days of culture.

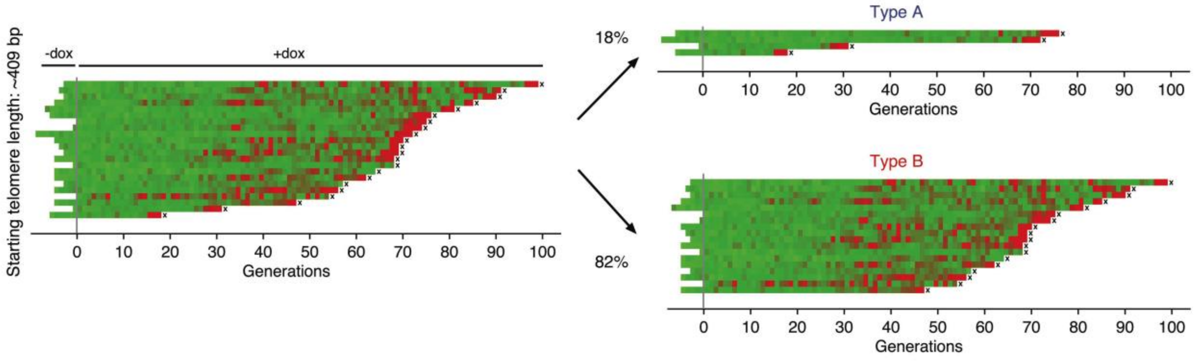

Supplementary Figure 8. Single-lineage analysis of the TetO2-*TLC1* strain (yT528) with elongated telomeres. The TetO2-*TLC1* strain was treated with ethanol to elongate the telomeres and allowed to recover in YPD for ~40 generations (Supplementary Fig. 7a, lanes 2 and 3). The cells were then injected into the microfluidic device for analysis of individual lineages (*n* = 22, doxycycline addition at generation 0). In parallel, a sample of the cells was grown for 12 h and then subjected to terminal restriction fragment Southern blotting to measure the average telomere length at the start of the single-lineage analysis (Supplementary Fig. 7a, lane 4). Number of divisions undergone after doxycycline addition: 69 ± 19 (median ± SD). The cells were clustered into type A and type B lineages and their proportions are indicated.

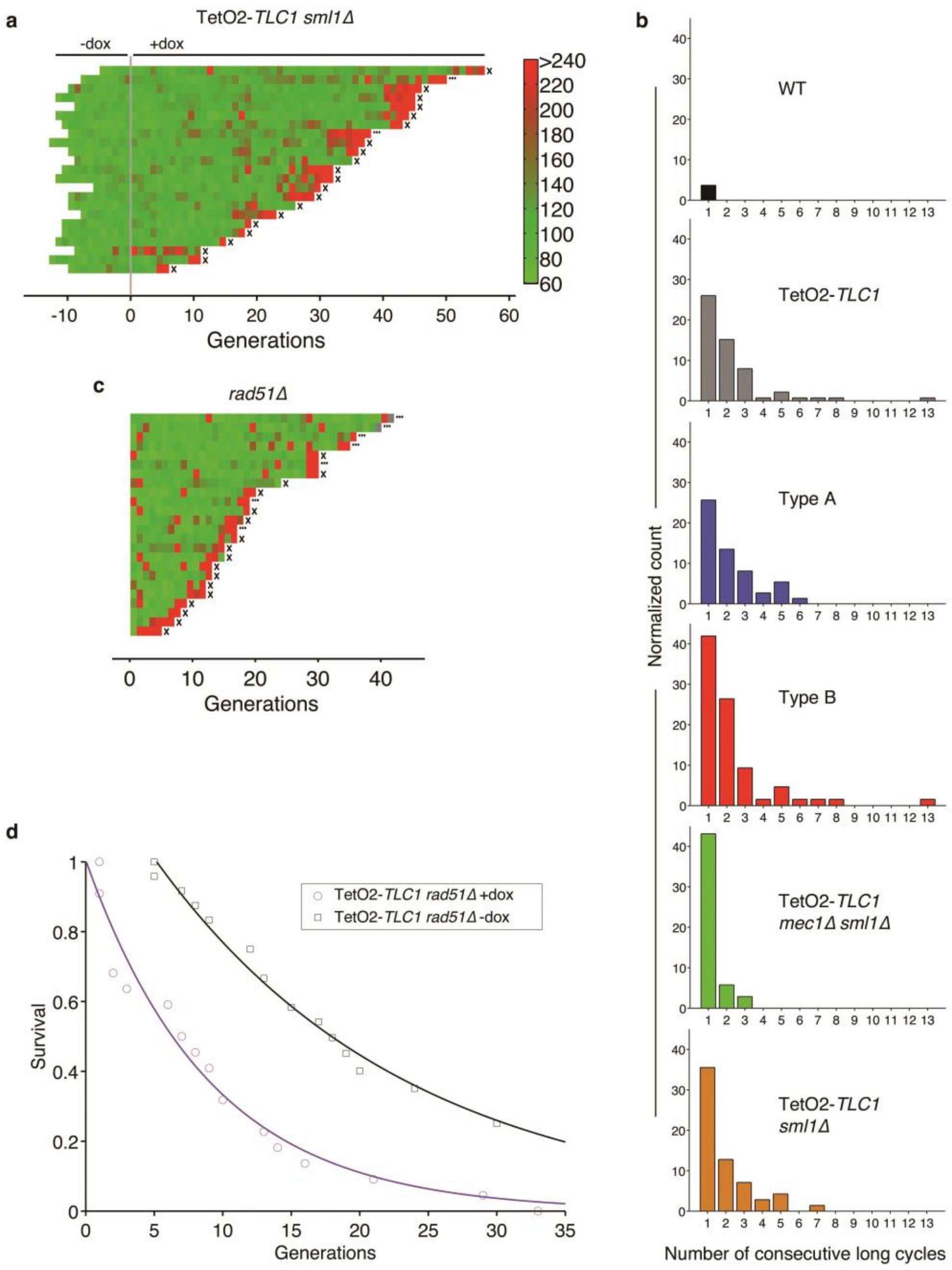

Supplementary Figure 9. Loss of Mec1 reduces consecutive cell-cycle arrests. a, The cell-cycle duration and generation number of telomerase-negative *sml1*Δ lineages (yT599, *n* = 23) are similar to those of telomerase-negative *SML1* lineages. **b,** Distribution of consecutive long cell cycles (defined as greater than the mean +3 SD of wild-type cell-cycle duration) in the indicated strains. Counts are normalised to the number of generations evaluated and are shown as counts per 1,000 generations. Consecutive long cycles are virtually absent from Mec1-deficient lineages. **c,** Telomerase-positive *rad51*Δ lineages (yT641, *n* = 24) have a high spontaneous rate of cell death. **d,** Fit of the survival curves for TetO2-*TLC1 rad51*Δ mutants (yT641) cultured with or without doxycycline. The curves were fitted with a decreasing exponential function $y = ae^{-bx}$, with a and b fitting parameters. b defines the mortality rate per division. When telomerase is active (black line), $a = 1.3$, $b = 0.054$, and $R^2 = 0.99$. When telomerase is inactivated (purple line), $= 1.0$, $b = 0.11$, and $R^2 = 0.97$.

Strain	Name	Genotype
yT538	Wild-type	*Matα ura3-1 trp1-1 leu2-3,112 his3-11,15 ADE2 LYS2 RAD5 cdc10::CDC10-mcherry-kanMX TLC1*
yT528	TetO2-*TLC1*	yT538 *tlc1::HIS3MX6*-PrTetO2-*TLC1*
yT598	TetO2-*TLC1 mec1Δ sml1Δ*	yT528 *mec1::kanMX2 sml1::HIS3*
yT599	TetO2-*TLC1 sml1Δ*	yT528 *sml1::HIS3*
yT641	TetO2-*TLC1 rad51Δ*	yT528 *rad51::LEU2*
yT639	*est2-D670A*	yT538 *est2::est2-D670A* complemented with pVL291(*EST2-URA3*)
yT662	TetO2-*TLC1 pol32Δ*	yT528 *pol32::LEU2*

Supplementary Table 1. List of strains used.
